# Genomic landscape of epithelium with low-grade atypia on gastric cancer after *Helicobacter pylori* eradiation therapy

**DOI:** 10.1007/s00535-019-01596-4

**Published:** 2019-06-13

**Authors:** Kazuhiko Masuda, Yuji Urabe, Masanori Ito, Atsushi Ono, Hayes Clair Nelson, Koki Nakamura, Takahiro Kotachi, Tomoyuki Boda, Shinji Tanaka, Kazuaki Chayama

**Affiliations:** 1 Department of Gastroenterology and Metabolism, Applied Life Science, Institute of Biomedical and Health Science, Hiroshima University, 1-2-3 Kasumi, Minami-ku, Hiroshima, 734-8551 Japan; 2 Department of Regeneration and Medicine Medical Center for Translation and Clinical Research, Hiroshima University Hospital, Hiroshima, Japan; 3 Department of Endoscopy, Hiroshima University Hospital, Hiroshima, Japan; 4 Research Center for Hepatology and Gastroenterology, Hiroshima University, Hiroshima, Japan

**Keywords:** Gastric cancer, Eradiation therapy for *Helicobacter pylori*, Epithelium with low-grade atypia, Laser microdissection

## Abstract

**Background:**

Gastric cancer may develop after successful eradication of *Helicobacter pylori,* although the incidence is lower than in non-eradicated individuals. We previously reported the appearance of characteristic epithelium with low-grade atypia (ELA) on the surface of gastric cancer after *H. pylori* eradication. However, whether ELA originates from cancer after re-differentiation or from the non-cancerous surrounding mucosa is unknown.

**Methods:**

We isolated ELA regions from 10 early gastric cancer patients and analyzed the nucleotide sequences for 90 oncogenes and 35 fusion oncogenes, comparing them with counterpart cancer tissue, normal gastric mucosa, and blood cell-derived DNA. Somatic mutations in each tissue were identified by comparing them with the sequences from whole blood-derived DNA.

**Result:**

Gene alterations were observed in nine of the ten patients, and up to 42 and 70 somatic mutations were seen in cancer and ELA samples, respectively. Common mutations shared between cancer and ELA tissues were found in eight of these nine patients. In contrast, common mutations between non-cancer mucosa and ELA were only detected in one patient, who also had common mutation between cancer and ELA. ELA-specific nucleotide substitutions were seen in seven patients. In contrast, cancer-specific substitutions were only found in two patients. 18 out of 19 amino acid substitutions present in cancer tissue were also identified in ELA. These results suggest that ELA originated from cancer tissue and accumulated further nucleotide substitutions.

**Conclusions:**

Differential diagnosis of ELA and normal mucosa should be carefully performed to prevent misdiagnosis of ELA as normal mucosa with atypia.

**Electronic supplementary material:**

The online version of this article (10.1007/s00535-019-01596-4) contains supplementary material, which is available to authorized users.

## Introduction

Gastric cancer (GC) is one of the most common types of cancer worldwide. It is the third leading cause of cancer-related death in both sexes worldwide [1]. East Asia, in particular, has a high incidence rate of stomach cancer [2]. The most common cause of gastric cancer is *Helicobacter pylori* (*H. pylori*), a Gram-negative microaerophilic bacterium [3]. The International Agency for Research on Cancer, a subordinate organization of the World Health Organization, identified *H. pylori* as a definite carcinogen in 1994 [4]. The effect of suppressing carcinogenesis by *H. pylori* eradication has been reported by various studies [5, 6]. After the relationship between *H. pylori* and gastric cancer was identified, the Japanese National Health Insurance system has covered the cost for *H. pylori* eradication therapy for patients with *H. pylori*-associated chronic gastritis since 2013.

Eradication of *H. pylori* is expected to prevent the development of gastric cancer; however, primary or metachronous gastric cancer was discovered in some patients following successful *H. pylori* eradication. Gastric cancer may still develop after successful eradication of *H. pylori,* although the incidence is lower than that of individuals in whom *H. pylori* has not been eradicated. Kamada et al. [7] reported an annual gastric cancer incidence rate of 0.24% in patients who had undergone successful eradication therapy. Characteristics of gastric cancer detected after successful eradication therapy were reported as follows: a lesion with diameter of typically < 20 mm, located in the middle and lower parts of the stomach, a depressed microscopic type, and a differentiated histology [8, 9]. Recently, it has been recognized that gastric cancer detected after *H. pylori* eradication is often difficult to diagnose by endoscopy because of its indistinct border or lack of obvious cancerous characteristics [10]. There are several causes for the difficulty of detecting early GC after *H. pylori* eradication, including morphological changes within the tumor, color changes of background mucosa, and histopathological changes in the surface area of GC.

We previously reported that characteristic epithelium with low-grade atypia (ELA) may appear on the surface of gastric cancer after *H. pylori* eradication [11, 12]. The presence of this epithelium makes it difficult to diagnose gastric cancer after eradication, as has been reported elsewhere [13, 14]. This epithelium mainly appears on the surface of differentiated-type cancer and shows no proliferating activity, with characteristics similar to that of normal foveolar epithelium [12]. Concerning the pathogenesis of ELA, we have considered the possibility that the malignant character of gastric cancer tissue may be restored after eradication therapy followed by the appearance of ELA. However, non-neoplastic glands can be found among the gastric cancer tissue and appear in the surface of the gastric cancer lesion. Thus, we cannot exclude the possibility that ELA is derived from these intermixed non-neoplastic gastric glands with regenerative or metaplastic changes. Essentially, it is unclear whether ELA lesions are neoplastic or non-neoplastic and whether adenocarcinoma tissue can be restored into ELA by the acquired event.

In the present study, we tried to confirm the etiology of ELA by performing deep target-sequencing to compare somatic mutation profiles in cancer, normal mucosa, and ELA tissues.

## Methods

### Patients and sample preparation

**Fig. 1:**
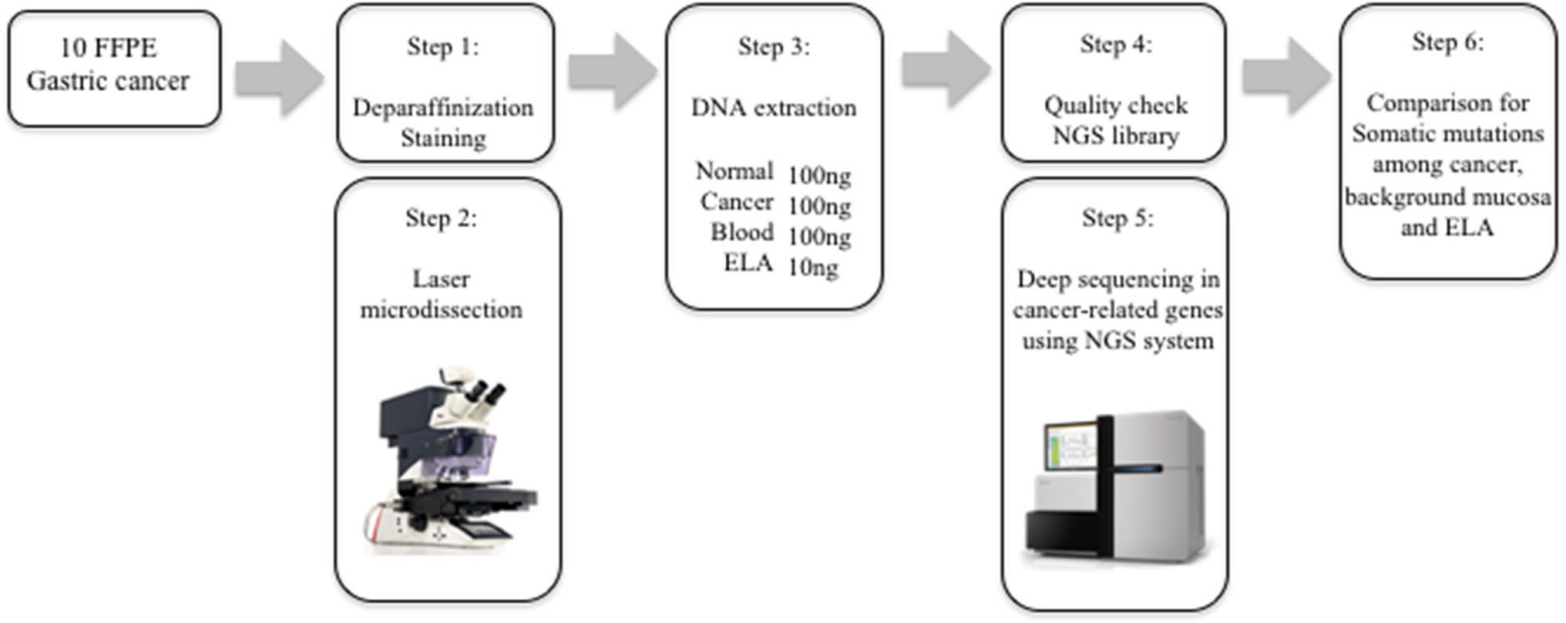
Experimental workflow

For this study, patients were selected and their tissue samples were extracted between June 2013 and September 2016 at Hiroshima University Hospital in Hiroshima, Japan. The study population consisted of 40 serial peripheral blood samples, matched tumor samples, normal gastric mucosa, and epithelium with low-grade atypia (ELA) present on the surface of gastric cancers from 10 patients with post-eradication early-stage GCs. Post-eradication GCs were defined as GCs detected in follow-up more than 1 year after *H. pylori*-eradication therapy. All post-eradication GCs were treated by endoscopic submucosal dissection (ESD). Clinicopathological characteristics of the patients in this study are shown in Table S1. Targeted next-generation sequencing (NGS) of DNA extracted from ELA, tumor tissues, normal gastric mucosa tissues, and control lymphocytes exhibited somatic rearrangements (Fig. 1). The Human Ethics Review Committee of Hiroshima University approved the study. All patients provided written informed consent.

## Histological definition of epithelium with low-grade atypia

**Fig. 2:**
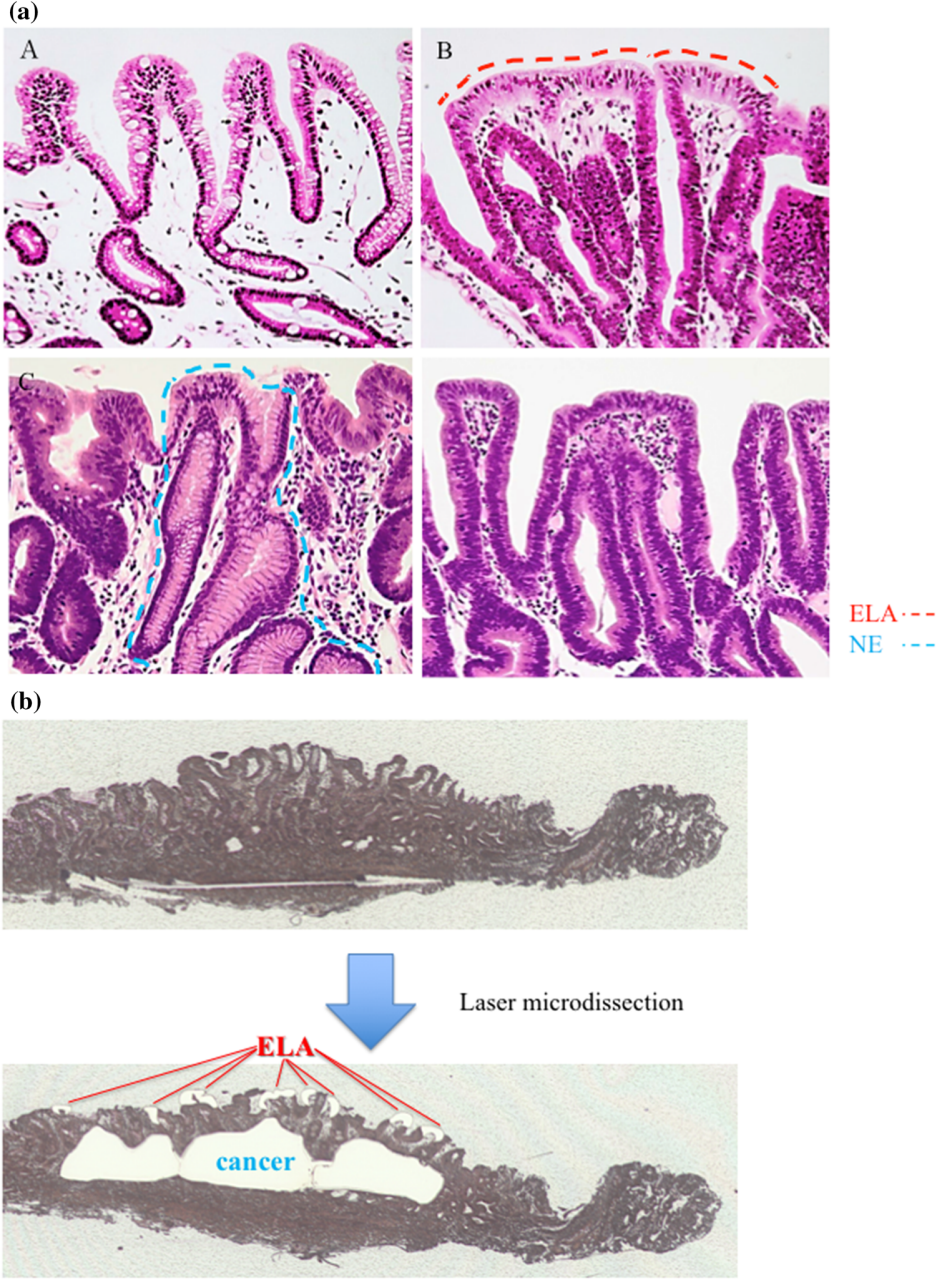
Extraction methods for cancerous tissue, normal tissue, and ELA pathological specimens were stained using Arcturus Paradise PLUS Reagent System (Thermo Fisher). The range of ELA was diagnosed by two pathologists who were familiar with the digestive tract. Tissue extraction was done by LMD 6000 (Leica, Germany). a; Representative image of each part in GC after *Hp* eradication. **a** Indicates normal epithelium. **b** is epithelium of low-grade atypia (ELA) covering the surface of gastric cancer tissue and is indicated by the red dotted line. **c** Is non-neoplastic epithelium (NE) found in the cancer area and indicated by the blue dotted line. **d** Is cancer tissue without ELA (**e** ×100). **b** Extraction methods of ELA and cancerous tissues by LMD as in figure

To evaluate ELA in each patient, two specialists (MI and KM) examined histological sections of endoscopically resected gastric cancer tissue after staining with hematoxylin and eosin (HE). ELA was defined according to the following criteria, as previously described [10]: (1) ELA must lie on the surface of gastric cancer tissue, (2) ELA must be columnar epithelium with spindle or oval nuclei, (3) nuclear polarity must be present in the ELA, and 4) the ELA must be separated and distinguished from the surrounding non-neoplastic mucosa. We cautiously excluded intermixed non-neoplastic glands from ELA lesions by histological evaluation with HE-stained sections (Fig. 2a).

## Tissue capturing and DNA extraction

Pathologic tumor tissues, normal gastric mucosa, and ELA were dissected from ten 10-μm-thick slides made from formalin-fixed paraffin-embedded (FFPE) specimens which were deparaffinized, stained, and dehydrated via the Arcturus Paradise PLUS Reagent System (Thermo Fisher Scientific, Waltham, MA, USA); dissections were performed via the Laser Capture Microdissection System (Leica LMD 6500) in accordance with pathogenesis diagnosis (Fig. 2a, b**)**. The DNA was extracted from these tissues using the GeneRead DNA FFPE Kit (Qiagen, Valencia, CA, USA), and concentrations were determined using the Qubit 1.0 Fluorometer (Life Technologies, Grand Island, NY, USA). The quantity and quality of the FFPE-derived DNA samples were checked by calculating the normalized DNA integrity scores (ΔΔCq) via qPCR analysis using the Agilent NGS FFPE QC Kit (Agilent, United States).

## Control lymphocytes

Seven milliliters of peripheral venous blood were collected from each of 10 post-eradication GC patients. The genomic DNA of lymphocytes was extracted and used as germline controls.

## Target enrichment and next-generation sequencing

DNA extracted from tumors, normal gastric mucosa, ELA, and normal lymphocytes was fragmented into 150–200 bp by sonication using a Covaris S2 (6 min, 10% duty, intensity = 5, 200 cycles/burst; Covaris Inc.) and used for library construction according to the manufacturer’s instructions. In all cases, 40 ng of DNA was prepared for sequencing. The exons of 90 oncogenes and the associated introns of 35 fusion oncogenes were enriched using the SureSelect-XT HS NCC oncopanel (Agilent, Table S2). The resulting pooled libraries were quality control-checked via the High Sensitivity D1000 ScreenTape System using the 2200 TapeStation Instrument (Agilent). Sequencing was performed with paired-end reads via the HiSeq 2500 platform (Illumina, USA).

## Variant detection

Sequencing reads were aligned to the hg19/GRCh37 reference sequence and analyzed using SureCall Software version 4 (Agilent Technologies, Santa Clara, CA, USA). PCR duplicates were removed by molecular barcode, which was performed using SureCall Software version 4 to improve mapping quality prior to variant calling. Paired-end and single analysis in SureCall Software version 4 were used to identify single nucleotide variants and insertions/deletions (indels) in tumors, normal gastric mucosa, and ELA. Called variants were considered germline mutations if they were also present in the control lymphocytes. To reduce the false-positive rate, we set the cutoff values for somatic mutations in tumors, normal gastric mucosa, and ELA as follows: read depth > 20 and forward/reverse balance between 0.25 and 0.75. We also configured the SureCall SNPi caller using SureSelect default settings: variant score threshold 0.3, minimum quality for base 30, variant call quality threshold 100, minimum allele frequency 0.1, minimum number of reads supporting variant allele 10. Moreover, variants that (a) were repeated sequences registered in UCSC'si repeat masker (b) called as replacements, or (c) were clearly identified as sequence errors in the Integrated Genomic Viewer (Broad Institute) were excluded as somatic mutation candidates in all sample types. We classified somatic mutations into three categories: Category (I) frameshift indels or nonsense mutations; Category (II) missense mutations; and Category (III) synonymous changes or mutations located at introns.

## Immunohistochemistry of p53

Paraffin-embedded human CRC tissue was cut into 2–3 μm sections and mounted on positively charged slides. Antigen retrieval was conducted with Tris–EDTA buffer (pH 9.0) in a microwave oven at 800 W for 5 min and at 150 W for 10 min. The slides were then incubated with a primary antibody. Anti-human p53 antibody (M3636; Dako, Tokyo, Japan) was applied at a dilution of 1:500 for 2 h; the antibody were applied at room temperature. The bound antibodies were detected using the EnVision system (Dako, Copenhagen, Denmark). After immunostaining, the slides were counterstained with hematoxylin.

### Statistical analysis

The number of mutations shared between cancer and ELA tissue was compared using paired *t* test. The differences in mutant allele frequency between tumors and matched ELAs were assessed using the Wilcoxon signed-rank test. Comparisons were considered significant if the P-value was less than 0.05. All statistical analyses were performed using R version 3.3.1.

## Results

### Quality and quantity of DNA extracted from tumor, normal, and ELA tissues

We succeeded in extracting and separating tissues from early-stage GCs, matched normal gastric mucosa, and ELA from endoscopic resected 1000–10,000 μm^2^ FFPE specimens in all 10 patients by LMD. We were able to extract 10–800 ng DNA from each type of tissue. An estimate of the amount of FFPE-derived genomic DNA degradation using the ⊿⊿*C*_q_ gave a median value of 0.4 (range 0.02–3.1), indicating that high-quality DNA had been extracted from the FFPE specimens.

## Identification of somatic mutations in tumor, normal, and ELA tissues

**Fig. 3:**
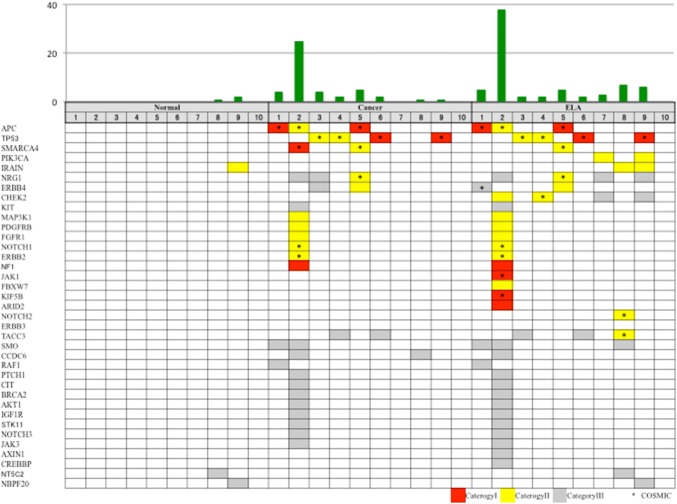
Mutation profiling of normal mucosa, cancerous tissues, and ELA in ten cases. The upper panel shows the number of somatic mutations in each tissue type for each of the ten cases. The lower panel shows the mutation pattern in each mutated gene in each tissue from the ten cases. Red cells represent Category I mutation (frameshift indels or nonsense mutations), yellow cells represent Category II mutations (missense mutations), and gray cells represent Category III mutations (synonymous mutations or mutations located within introns). Asterisks represent mutations found in the COSMIC database

After removal of PCR duplicates, the depth of the sequencing coverage in target regions was in the range of 60–423 (Table S3), and the median percentage of reads mapped to the target area was 77.1–96.9% (Table S3). In 8 out of 10 cases, between one and 27 somatic mutations per patient were identified in tumor tissues (Fig. 3, Table S4). Out of 42 somatic mutations derived from cancer tissues, 8 (19.0%) were classified as Category I (5 frameshift indels and 3 nonsense mutations), 10 (23.8%) were Category II (missense mutations), and 24 (57.1%) were Category III (Table S4). On the other hand, somatic mutations were found in normal tissues in two of the patients, two in one patient and one in the other (Table S4). Out of these three somatic mutations observed in normal tissues, one (33.3%) was Category II, and two (66.6%) were Category III (sequence depth was in the range of 164–423, and the median percentage of reads mapped to the target area was 96%; 90.1–96.9%)( Fig. 3, Table S3, S4). The 19 nonsynonymous mutations in tumor tissues were located in APC (mutated in 31% of our samples), TP53 (16%), SMARC4 (10%), ERBB2 (5.2%), FGFR1 (5.2%), MAK3K1 (5.2%), NOTCH1(5.2%), PTGFRB (5.2%), ERBB4 (5.2%), NRG1 (5.2%), SMARC4 (5.2%), and NF1 (5.2%) (Fig. 3, Table S4).

Nine out of the 10 ELA samples had between one and 38 somatic mutations (Fig. 3, Table S4). After removal of PCR duplicates, the coverage depth of ELA samples in the target regions was in the range of 60–244, and the median percentage of reads that mapped to the target area was 86% (77–93%) (Table S3). Out of 70 somatic mutations found in ELA tissues, 10 (14.2%) were Category I (7 frameshift indels and 3 nonsense mutations), 22 (28.5%) were Category II (missense mutations), and 38 (54.2%) were Category III (Table S4).

We uploaded the sequence data into the NBDC Human Database (https://humandbs.biosciencedbc.jp) and will include the assigned accession number.

## Phylogenetic trees of somatic mutations

**Fig. 4:**
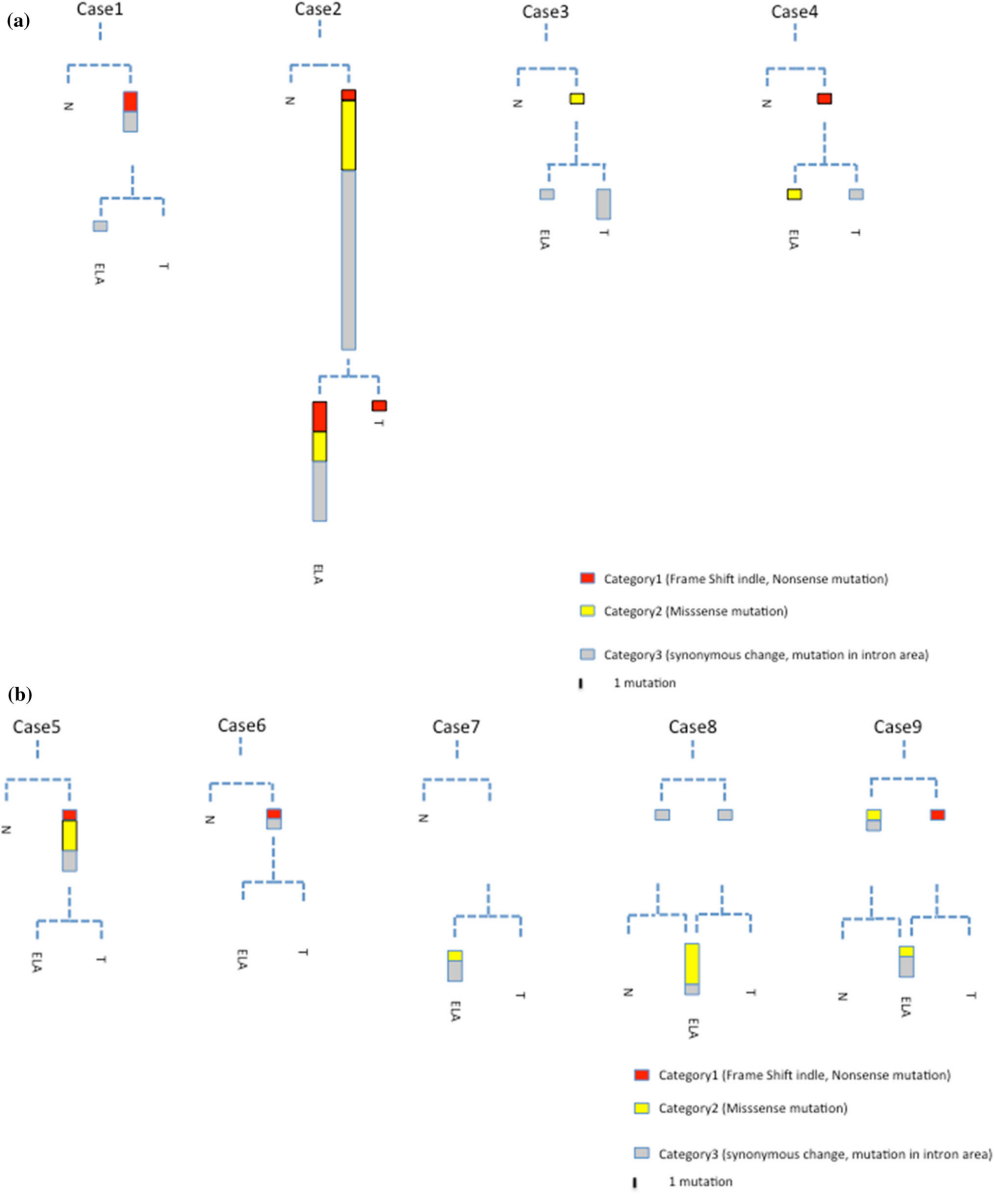
Phylogenetic trees of somatic mutations in cancerous tissue, normal mucosal tissue, and ELA tissue for each sample. The branch length reflects the count of somatic mutations. Red color represents nonsense mutations or frameshift indels. Yellow represents missense mutations. Black represents synonymous mutations and mutations within introns. a; Case1-4, b; Case 5–9

Using these catalogues of somatic mutations in normal, cancer, and ELA tissues, we derived phylogenetic trees for each individual (Fig. 4). The structure of the trees recapitulated the spatial orientation of ELA within each case, with more closely related branches originating from the cancer tissue: for example, in cancer tissues from cases 1, 2, 5, 6, 8 and 9, each of the somatic mutations found in the cancer tissue was also found in ELA tissues extracted from the same patient. Similarly, in cases 1, 3, 4, 5, and 6, each of the nonsynonymous mutations found in the cancer tissue was also present in the ELA tissue from the same patient (Fig. 4). Furthermore, in case 2, mutation profiling of ELA indicated a similar pattern of hypermutation to that of cancer tissue. These results suggest that ELA originated from the cancer tissue in these patients.

## Mutation load in normal, cancer and ELA tissues

We found no somatic mutations in common among normal, cancer and ELA tissues in any patient (Fig. 4, supplementary table 3). While we found only three somatic mutations (3/70 4.2%) derived from normal tissues, we found 46 cancer-derived somatic mutations (46/70. 65.7%) that were also present in ELA tissue (Fig. 4, Table S4). Moreover, 18 out of 19 nonsynonymous mutations found in cancer tissues were also detected in ELA tissue (Figs. 3, 4, Table S4). In addition to the large number of somatic mutations shared between cancer and ELA tissues, the number of somatic mutations in ELA tissues was larger than that of cancer tissues **(**median: 2 vs 5, p = 0.084**,** Fig. 3, Table S4). Additional distinct somatic mutations were found in ELA tissue, suggesting that ELA developed following the occurrence of cancer.

### Comparison of MAF between ELA and cancer tissue

It is impossible to completely separate ELA and cancer tissues even with the use of LMD. Therefore, to study the small amount of contamination between cancer and ELA tissues, we focused on differences in the mutant allele fraction (MAF) of somatic mutations between ELA and cancer tissues. If somatic mutations detected in the ELA samples originated due to contamination with a small amount of cancer tissue, the MAF of the somatic mutations in the ELA samples should be much lower than in each of the corresponding somatic mutations in cancer samples. However, there was no difference in the MAF of somatic mutations between cancer and ELA tissues (Figure S1). Furthermore, the MAFs of somatic mutations in cancer tissues were mostly equal or higher in paired ELA samples, suggesting that the origin of ELA is from cancer tissues (Figure S1).

### Immnohistochemistry of p53

We examined a p53 protein to check whether the mutational status was consistent with protein level by immunohistochemical analysis. However, as demonstrated in our previous paper [12], p53 overexpression was not found in ELA tissue.

## Discussion

In this study, using NGS analysis, we clarified the origin of the characteristic epithelium with low-grade atypia referred to as ELA that appears on the surface of gastric cancer after *H. pylori* eradication therapy. Some studies have described ELA mainly as a remnant or as regenerated normal gastric glands intermixed with gastric cancer tissue or as expansion of normal mucosa surrounding cancer tissues [13, 14]. It should be emphasized that these non-neoplastic tissues can be clearly distinguished from ELA in our study. However, regenerative or metaplastic changes may also be evident in this non-neoplastic epithelium, resulting in complicated histological features. Recently, Nimura et al. reported that this ELA-like characteristic feature detected in the surface of gastric cancer was characteristically associated with successful eradication therapy and suggested that this histological lesion should be called “non-neoplastic epithelium” [15].

We concluded that ELA was derived from gastric cancer tissue and may have resulted from the restoration of malignant characteristics following the eradication of *H. pylori*. The somatic mutations found in ELA were identical to those in matched gastric cancer tissues from the same patient, strongly suggesting that ELA derives from cancer tissue. Moreover, it has been suggested that the morphology of human adenocarcinoma tissue can be altered to low-grade malignancy via eradication therapy. Recently, Dow et al. demonstrated that colon adenocarcinoma could revert to normal gland tissue by restoration of a key gene in an animal model [16]. In this study, we did not observe restoration of any somatic mutations from cancerous tissue to ELA. However, it is possible that the affected genes were not included in the cancer panel used in this study. In addition, epigenomic alterations may contribute to the restoration of gastric tumorigenesis. Previous studies have demonstrated that methylation levels in gastric epithelial cells diminished dramatically following *H. pylori* eradication [17].

After eradication therapy, various environmental alterations were induced in the stomach as well as in the surrounding cancer tissue. Previous studies have demonstrated that gastric cancer detected after eradication therapy shows some characteristic features, such as reduced proliferation activity [18] and induced expression of gastric mucin phenotype [19, 20]. It is notable that serum gastrin levels have been reported to decrease following eradication therapy [21]. Gastrin is a well-known growth-promoting factor for gastric cancer cells, and our previous report demonstrated that the gastrin receptor was detected in gastric cancer cells [22]. Previously, we reported that the gastrin–gastrin receptor system is implicated in the morphological changes induced by eradication therapy [23]. We hypothesize that diminished gastrin levels may contribute to the restoration of malignant morphology of gastric cancer tissue, including the appearance of ELA. However, the main reason for this phenomenon remains unclear.

Other factors, including increased gastric acid or reduced inflammatory cytokines, may influence this morphological conversion. Gastric acid is widely known as an important feature of the tumor microenvironment and a major determinant of tumor progression [24-27]. In addition, chronic inflammation plays a critical role in cancer prognosis. Hibiya et al. reported that chronic inflammation led to sustained activation of NF-κB signaling in colonic organoids, resulting in cell transformation that might be related to the carcinogenesis of CAC in UC [28].

This study has several limitations, the most crucial of which is the small number of patients enrolled in the study. However, all mutations in eight out of ten carcinoma tissues matched those in ELA, and there was little possibility of carcinoma tissue contamination into ELA sample based on MAF results. We excluded false-positive variants in sequence data as accurately as possible by excluding suspected sequencing errors. Moreover, variants that were called in cancer and ELA tissues but that were also called with low frequencies in normal tissue were excluded by IVG viewer. The next limitation is that, given the data we collected for this study, we were unable to determine the cause of gene alteration in ELA induced by eradication therapy, and it still remains unknown. We hypothesize that increased gastric acid secretion may be a cause of gene alteration, and we have recently begun to investigate the effect of acidic conditions on gene alteration in vitro. Alteration of intra-gastric bacterial flora may be another cause of gene alteration induced by eradication therapy. Ushijima et al. reported that methylation levels in gastric mucosa recover via HP eradication therapy [29, 30]. Thus, it is possible that the development of ELA was associated with epigenetic changes resulting from therapy to eradicate *Helicobacter pylori* from the gastric mucosa. Therefore, the next step will be to examine epigenetic changes associated with the progression from cancer tissue to ELA by whole-transcriptome sequencing and whole-genome bisulfite sequencing. The final main limitation of this study is that the input DNA volume from ELA tissue used the in preparation of the sequence library is less than that of the other tissues. The area of ELA per tumor is very small compared to cancerous tissue. Therefore, only 10 ng of DNA could be extracted from ELA, which is the minimum volume to successfully sequence the library. As a result, however, PCR duplicates occur at a higher frequency in sequence libraries containing only the minimum volume of DNA. Consequently, we used the molecular barcode method [31], to exclude sequencing errors due to low input DNA volume.

In summary, we discovered that ELA derives from cancer tissue. Gastric cancer tissue can convert to low-grade atypical tissue by eradication therapy. The results of this study emphasize the critical role of molecular imaging during post-eradication gastric cancer.

## Electronic supplementary material

Below is the link to the electronic supplementary material.

Supplementary file1 (TIFF 1521 kb)

Supplementary file2 (DOCX 48 kb)

Supplementary file3 (DOCX 82 kb)

Supplementary file4 (DOCX 54 kb)

Supplementary file5 (DOCX 135 kb)
