# Supplementary figures and images for: Genomic landscape of epithelium with low-grade atypia on gastric cancer after Helicobacter pylori eradiation therapy
Source: J Gastroenterol. 2019 Jun 13;54(10):907–15. doi: 10.1007/s00535-019-01596-4 (PMC6759680; doi:10.1007/s00535-019-01596-4)

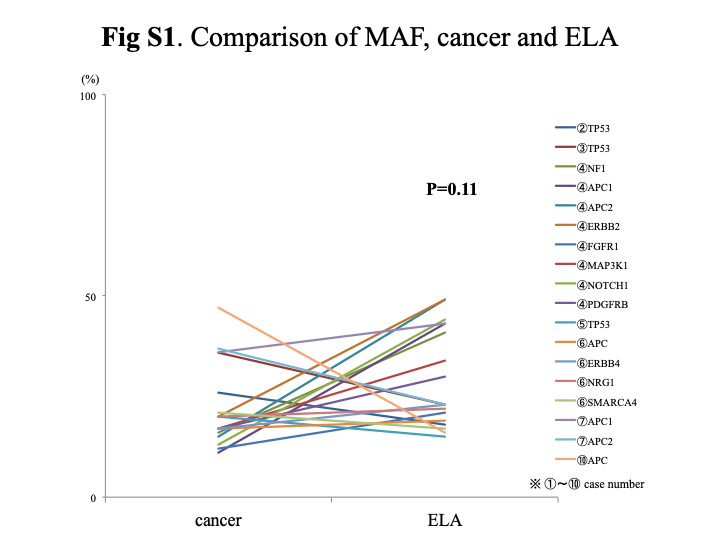

Supplement: Supplementary file 1 — Supplementary file1 (TIFF 1521 kb) [file 535_2019_1596_MOESM1_ESM.tiff]
