# Supplementary material for: Genomic landscape of epithelium with low-grade atypia on gastric cancer after Helicobacter pylori eradiation therapy
Source: J Gastroenterol. 2019 Jun 13;54(10):907–15. doi: 10.1007/s00535-019-01596-4 (PMC6759680; doi:10.1007/s00535-019-01596-4)
Supplement: Supplementary file 2 — Supplementary file2 (DOCX 48 kb) [file 535_2019_1596_MOESM2_ESM.docx]

**Table S1.**  Histological and endoscopic characteristics.

| Case | Histological type, predominant | Macroscopic type | Depth of invasion |
| --- | --- | --- | --- |
| 1 | tub1 | 0–IIa | Mucosal |
| 2 | tub1 | 0–IIc | Mucosal |
| 3 | tub1 | 0–IIa | Mucosal |
| 4 | tub1 | 0–IIc | Mucosal |
| 5 | tub1 | 0–IIc | Mucosal |
| 6 | tub1 | 0–IIc | Mucosal |
| 7 | tub1 | 0–IIc | Mucosal |
| 8 | tub1 | 0–IIa | Mucosal |
| 9 | tub1/por2 | 0–IIc | Mucosal |
| 10 | tub1 | 0–IIc | Mucosal |
