# Supplementary material for: Genomic landscape of epithelium with low-grade atypia on gastric cancer after Helicobacter pylori eradiation therapy
Source: J Gastroenterol. 2019 Jun 13;54(10):907–15. doi: 10.1007/s00535-019-01596-4 (PMC6759680; doi:10.1007/s00535-019-01596-4)
Supplement: Supplementary file 3 — Supplementary file3 (DOCX 82 kb) [file 535_2019_1596_MOESM3_ESM.docx]

**Table S2.** Oncogenes and fusion genes included in the SureSelect NCC oncopanel.

| **Oncogenes (Sequenced Exon Area)** | | | | | **fusion genes (Sequenced Intron Area)** | | |
| --- | --- | --- | --- | --- | --- | --- | --- |
| ABL1 | CREBBP | IGF1R | NOTCH1 | ROS1 | ALK | AGTRAP | TPM3 |
| AKT1 | CTNNB1 | IGF2 | NOTCH2 | SETD2 | AKT3 | C2orf44 | TPM4 |
| AKT2 | CUL3 | IL7R | NOTCH3 | SMAD4 | BRAF | CCDC6 | VCL |
| AKT3 | DDR2 | JAK1 | NRAS | SMARCA4 | EGFR | CD74 |  |
| ALK | EGFR | JAK2 | NRG1 | SMO | ERBB4 | CIT |  |
| APC | ENO1 | JAK3 | NT5C2 | STAT3 | FGFR2 | EML4 |  |
| ARID1A | EP300 | KEAP1 | PALB2 | STK11 | FGFR3 | EPB41 |  |
| ARID2 | ERBB2 | KIT | PBRM1 | TP53 | NOTCH1 | ESRP1 |  |
| ATM | ERBB3 | KRAS | PDGFRA | TSC1 | NRG1 | EZR |  |
| AXIN1 | ERBB4 | MAP2K1 | PDGFRB | VHL | RAF1 | FN1 |  |
| BAP1 | EZH2 | MAP2K4 | PIK3CA |  | RET | GOPC |  |
| BARD1 | FBXW7 | MAP3K1 | PIK3R1 |  | ROS1 | KIAA1549 |  |
| BCL2L11 | FGFR1 | MAP3K4 | PTCH1 |  |  | KIF5B |  |
| BRAF | FGFR2 | MDM2 | PTEN |  |  | KLC1 |  |
| BRCA1 | FGFR3 | MET | RAC1 |  |  | MAGI3 |  |
| BRCA2 | FGFR4 | MTOR | RAC2 |  |  | SDC4 |  |
| CCND1 | FLT3 | MYC | RAD51C |  |  | SEC16A |  |
| CDK4 | HRAS | MYCN | RAF1 |  |  | SLC34A2 |  |
| CDKN2A | IDH1 | NF1 | RB1 |  |  | SLC45A3 |  |
| CHEK2 | IDH2 | NFE2L2 | RET |  |  | TACC3 |  |
