# Supplementary material for: Genomic landscape of epithelium with low-grade atypia on gastric cancer after Helicobacter pylori eradiation therapy
Source: J Gastroenterol. 2019 Jun 13;54(10):907–15. doi: 10.1007/s00535-019-01596-4 (PMC6759680; doi:10.1007/s00535-019-01596-4)
Supplement: Supplementary file 4 — Supplementary file4 (DOCX 54 kb) [file 535_2019_1596_MOESM4_ESM.docx]

**Table S3** Coverage depth of the data from cancer panels.

|  |  | Sequence Quality | | |
| --- | --- | --- | --- | --- |
| case | Sample | Duplicate molecules % | Covered regions % | Depth in target regions |
| 1 | normal | 71.75 | 96.6 | 400 |
| 2 | normal | 90.09 | 90.13 | 164 |
| 3 | normal | 72.26 | 96.69 | 423 |
| 4 | normal | 75.81 | 93.38 | 339 |
| 5 | normal | 75.28 | 96.27 | 361 |
| 6 | normal | 73.38 | 96.86 | 376 |
| 7 | normal | 87.32 | 93.98 | 181 |
| 8 | normal | 75.33 | 96.3 | 387 |
| 9 | normal | 77.23 | 96.06 | 316 |
| 10 | normal | 71.23 | 96.97 | 377 |
|  | Mean±SD | 75.3±6.17 | 96.28±2.12 | 368.5±84.88 |
| 1 | cancer | 68.12 | 90.8 | 293 |
| 2 | cancer | 68.9 | 95.5 | 312 |
| 3 | cancer | 73.62 | 96.62 | 410 |
| 4 | cancer | 75.28 | 96.66 | 353 |
| 5 | cancer | 74.78 | 96.66 | 361 |
| 6 | cancer | 68.48 | 96.64 | 332 |
| 7 | cancer | 86.06 | 94.32 | 178 |
| 8 | cancer | 68.51 | 96.77 | 395 |
| 9 | cancer | 73.05 | 96.4 | 374 |
| 10 | cancer | 69.8 | 96.9 | 357 |
|  | Mean±SD | 71.42±5.19 | 96.63±1.80 | 355±62.57 |
| 1 | ELA | 94.75 | 85.45 | 130 |
| 2 | ELA | 96.49 | 81.77 | 88 |
| 3 | ELA | 94.92 | 86.36 | 129 |
| 4 | ELA | 95.82 | 86.82 | 99 |
| 5 | ELA | 93.75 | 90.46 | 80 |
| 6 | ELA | 95.26 | 88.56 | 120 |
| 7 | ELA | 96.92 | 79.03 | 60 |
| 8 | ELA | 89.24 | 77.08 | 187 |
| 9 | ELA | 91.54 | 86.81 | 208 |
| 10 | ELA | 89.69 | 93 | 244 |
|  | Mean±SD | 94.83±2.6 | 86.58±4.7 | 124.5±56.87 |
| 1 | blood | 68.55 | 96.01 | 215 |
| 2 | blood | 64.29 | 96.61 | 209 |
| 3 | blood | 63.92 | 96.52 | 204 |
| 4 | blood | 65.94 | 96.22 | 205 |
| 5 | blood | 66.2 | 95.99 | 265 |
| 6 | blood | 68.43 | 95.97 | 231 |
| 7 | blood | 67.45 | 96.11 | 221 |
| 8 | blood | 57.31 | 96.44 | 124 |
| 9 | blood | 62.34 | 96.37 | 209 |
| 10 | blood | 59.52 | 96.5 | 156 |
|  | Mean±SD | 65.11±3.56 | 96.29±0.23 | 209±36.89 |
