# Supplementary material for: Genomic landscape of epithelium with low-grade atypia on gastric cancer after Helicobacter pylori eradiation therapy
Source: J Gastroenterol. 2019 Jun 13;54(10):907–15. doi: 10.1007/s00535-019-01596-4 (PMC6759680; doi:10.1007/s00535-019-01596-4)
Supplement: Supplementary file 5 — Supplementary file5 (DOCX 135 kb) [file 535_2019_1596_MOESM5_ESM.docx]

**Table S4.** Location of identified genes and allele frequency of FFPE samples.

| Case. | Region | Gene | Chr. | Position | Ref. | mut. | function | Count | Coverage | MAF | COSMIC | Category | Origin |
| --- | --- | --- | --- | --- | --- | --- | --- | --- | --- | --- | --- | --- | --- |
| 1 | Cancer | RAF1 | 3 | 12641630 | G | A | SNV | 0.322 | 91 | 283 |  | Category III | ELA |
| 1 | Cancer | APC | 5 | 112170647 | G | A | SNV | 0.355 | 33 | 93 |  | Category I | ELA |
| 1 | Cancer | APC | 5 | 112175642 | G | T | SNV | 0.368 | 50 | 136 | p.E1451fs*23 | Category I | ELA |
| 1 | Cancer | SMO | 7 | 128851226 | G | T | SNV | 0.308 | 37 | 120 |  | Category III | ELA |
| 1 | ELA | ERBB4 | 2 | 212248574 | C | T | SNV | 0.114 | 10 | 88 | p.E1231D | Category III |  |
| 1 | ELA | RAF1 | 3 | 12641630 | G | A | SNV | 0.193 | 21 | 109 |  | Category III | T |
| 1 | ELA | APC | 5 | 112170647 | G | A | SNV | 0.432 | 19 | 44 |  | Category I | T |
| 1 | ELA | APC | 5 | 112175642 | G | T | SNV | 0.225 | 16 | 71 | p.E1451fs*23 | Category I | T |
| 1 | ELA | SMO | 7 | 128851226 | G | T | SNV | 0.271 | 19 | 70 |  | Category III | T |
| 2 | Cancer | KIT | 4 | 55524260 | C | T | SNV | 0.187 | 144 | 770 |  | Category III | ELA |
| 2 | Cancer | MAP3K1 | 5 | 56171013 | C | T | SNV | 0.174 | 195 | 1122 |  | Category II | ELA |
| 2 | Cancer | APC | 5 | 112175166 | C | T | SNV | 0.112 | 20 | 179 | p.T1292M | Category II | ELA |
| 2 | Cancer | APC | 5 | 112176192 | C | T | SNV | 0.151 | 83 | 548 | p.P1634L | Category II | ELA |
| 2 | Cancer | PDGFRB | 5 | 149502734 | C | T | SNV | 0.17 | 143 | 843 |  | Category II | ELA |
| 2 | Cancer | SMO | 7 | 128829100 | G | A | SNV | 0.183 | 36 | 197 |  | Category III | ELA |
| 2 | Cancer | NRG1 | 8 | 32539709 | A | G | SNV | 0.22 | 29 | 132 |  | Category III | ELA |
| 2 | Cancer | NRG1 | 8 | 32556137 | C | T | SNV | 0.149 | 88 | 591 |  | Category III | ELA |
| 2 | Cancer | NRG1 | 8 | 32556555 | A | G | SNV | 0.151 | 47 | 312 |  | Category III | ELA |
| 2 | Cancer | FGFR1 | 8 | 38271151 | G | A | SNV | 0.121 | 146 | 1207 |  | Category II | ELA |
| 2 | Cancer | FGFR1 | 8 | 38277156 | C | T | SNV | 0.104 | 102 | 984 |  | Category III | ELA |
| 2 | Cancer | FGFR1 | 8 | 38279474 | G | A | SNV | 0.107 | 24 | 224 |  | Category III | ELA |
| 2 | Cancer | PTCH1 | 9 | 98209509 | C | A | SNV | 0.181 | 138 | 763 |  | Category III | ELA |
| 2 | Cancer | NOTCH1 | 9 | 139396885 | C | G | SNV | 0.18 | 166 | 921 |  | Category III | ELA |
| 2 | Cancer | NOTCH1 | 9 | 139396893 | C | T | SNV | 0.133 | 123 | 923 | p.Y1738_V1739ins11 | Category II | ELA |
| 2 | Cancer | CCDC6 | 10 | 61643693 | T | C | SNV | 0.165 | 94 | 569 |  | Category III | ELA |
| 2 | Cancer | CCDC6 | 10 | 61659348 | G | A | SNV | 0.221 | 202 | 913 |  | Category III | ELA |
| 2 | Cancer | CIT | 12 | 120186656 | C | T | SNV | 0.163 | 83 | 508 |  | Category III | ELA |
| 2 | Cancer | BRCA2 | 13 | 32905082 | T | C | SNV | 0.207 | 18 | 87 |  | Category III | ELA |
| 2 | Cancer | AKT1 | 14 | 105237217 | C | T | SNV | 0.126 | 68 | 539 |  | Category III | ELA |
| 2 | Cancer | IGF1R | 15 | 99250897 | C | A | SNV | 0.147 | 138 | 937 | p.A67A | Category III | ELA |
| 2 | Cancer | ERBB2 | 17 | 37879658 | G | A | SNV | 0.195 | 171 | 878 | p.R678Q | Category II | ELA |
| 2 | Cancer | NF1 | 17 | 29552143 | CT | C | Indel | 0.164 | 99 | 603 |  | Category I | ELA |
| 2 | Cancer | SMARCA4 | 19 | 11141497 | TG | T | Indel | 0.159 | 110 | 691 | p.L1161fs*3 | Category I |  |
| 2 | Cancer | STK11 | 19 | 1218510 | C | T | SNV | 0.137 | 48 | 350 |  | Category III | ELA |
| 2 | Cancer | NOTCH3 | 19 | 15281555 | G | A | SNV | 0.118 | 71 | 600 |  | Category III | ELA |
| 2 | Cancer | JAK3 | 19 | 17946007 | G | A | SNV | 0.176 | 118 | 671 |  | Category III | ELA |
| 2 | ELA | JAK1 | 1 | 65325832 | C | CG | Indel | 0.336 | 49 | 146 | p.L431fs*22 | Category I |  |
| 2 | ELA | KIT | 4 | 55524260 | C | T | SNV | 0.365 | 42 | 115 |  | Category III | T |
| 2 | ELA | KIT | 4 | 55564641 | C | T | SNV | 0.124 | 11 | 89 |  | Category II |  |
| 2 | ELA | FBXW7 | 4 | 153268174 | G | A | SNV | 0.107 | 13 | 121 |  | Category II |  |
| 2 | ELA | MAP3K1 | 5 | 56171013 | C | T | SNV | 0.345 | 59 | 171 |  | Category II | T |
| 2 | ELA | APC | 5 | 112175166 | C | T | SNV | 0.426 | 26 | 61 | p.T1292M | Category II | T |
| 2 | ELA | APC | 5 | 112176192 | C | T | SNV | 0.485 | 63 | 130 | p.P1634L | Category II | T |
| 2 | ELA | PDGFRB | 5 | 149502734 | C | T | SNV | 0.299 | 44 | 147 |  | Category II | T |
| 2 | ELA | SMO | 7 | 128829100 | G | A | SNV | 0.37 | 17 | 46 |  | Category III | T |
| 2 | ELA | SMO | 7 | 128846293 | T | A | SNV | 0.134 | 11 | 82 |  | Category III |  |
| 2 | ELA | NRG1 | 8 | 32410829 | T | C | SNV | 0.216 | 16 | 74 |  | Category III |  |
| 2 | ELA | NRG1 | 8 | 32489326 | C | A | SNV | 0.228 | 36 | 158 |  | Category III |  |
| 2 | ELA | NRG1 | 8 | 32513189 | C | T | SNV | 0.281 | 27 | 96 |  | Category III |  |
| 2 | ELA | NRG1 | 8 | 32539709 | A | G | SNV | 0.38 | 27 | 71 |  | Category III | T |
| 2 | ELA | NRG1 | 8 | 32556137 | C | T | SNV | 0.218 | 37 | 170 |  | Category III | T |
| 2 | ELA | NRG1 | 8 | 32556555 | A | G | SNV | 0.233 | 24 | 103 |  | Category III | T |
| 2 | ELA | FGFR1 | 8 | 38271151 | G | A | SNV | 0.213 | 54 | 254 |  | Category II | T |
| 2 | ELA | FGFR1 | 8 | 38277156 | C | T | SNV | 0.171 | 38 | 222 |  | Category III | T |
| 2 | ELA | FGFR1 | 8 | 38279474 | G | A | SNV | 0.127 | 14 | 110 |  | Category III | T |
| 2 | ELA | PTCH1 | 9 | 98209509 | C | A | SNV | 0.366 | 52 | 142 |  | Category III | T |
| 2 | ELA | NOTCH1 | 9 | 139395185 | G | A | SNV | 0.331 | 51 | 154 |  | Category II |  |
| 2 | ELA | NOTCH1 | 9 | 139396885 | C | G | SNV | 0.255 | 24 | 94 |  | Category III | T |
| 2 | ELA | NOTCH1 | 9 | 139396893 | C | T | SNV | 0.44 | 37 | 84 | p.Y1738_V1739ins11 | Category II | T |
| 2 | ELA | KIF5B | 10 | 32311825 | AT | A | Indel | 0.197 | 23 | 117 | p.M622fs*8 | Category I |  |
| 2 | ELA | CCDC6 | 10 | 61643693 | T | C | SNV | 0.392 | 67 | 171 |  | Category III | T |
| 2 | ELA | CCDC6 | 10 | 61659348 | G | A | SNV | 0.429 | 88 | 205 |  | Category III | T |
| 2 | ELA | ARID2 | 12 | 46287282 | GCT | G | Indel | 0.274 | 29 | 106 |  | Category I |  |
| 2 | ELA | CIT | 12 | 120186656 | C | T | SNV | 0.363 | 41 | 113 |  | Category III | T |
| 2 | ELA | BRCA2 | 13 | 32905082 | T | C | SNV | 0.3 | 15 | 50 |  | Category III | T |
| 2 | ELA | AKT1 | 14 | 105237217 | C | T | SNV | 0.33 | 35 | 106 |  | Category III | T |
| 2 | ELA | IGF1R | 15 | 99250897 | C | A | SNV | 0.358 | 48 | 134 | p.A67A | Category III | T |
| 2 | ELA | AXIN1 | 16 | 347014 | C | T | SNV | 0.184 | 14 | 76 |  | Category III |  |
| 2 | ELA | CREBBP | 16 | 3830884 | G | T | SNV | 0.141 | 13 | 92 |  | Category III |  |
| 2 | ELA | ERBB2 | 17 | 37879658 | G | A | SNV | 0.489 | 114 | 233 | p.R678Q | Category II | T |
| 2 | ELA | NF1 | 17 | 29552143 | CT | C | Indel | 0.41 | 55 | 134 | p.L1161fs*3 | Category I | T |
| 2 | ELA | STK11 | 19 | 1218510 | C | T | SNV | 0.365 | 42 | 115 |  | Category III | T |
| 2 | ELA | NOTCH3 | 19 | 15281555 | G | A | SNV | 0.323 | 42 | 130 |  | Category III | T |
| 2 | ELA | JAK3 | 19 | 17946007 | G | A | SNV | 0.347 | 50 | 144 |  | Category III | T |
| 3 | Cancer | ERBB4 | 2 | 212492542 | G | T | SNV | 0.116 | 34 | 293 |  | Category III |  |
| 3 | Cancer | NRG1 | 8 | 32520391 | T | C | SNV | 0.167 | 42 | 252 |  | Category III |  |
| 3 | Cancer | NRG1 | 8 | 32530077 | T | C | SNV | 0.151 | 18 | 119 |  | Category III |  |
| 3 | Cancer | TP53 | 17 | 7577120 | C | T | SNV | 0.256 | 90 | 351 | p.R273H | Category II | ELA |
| 3 | ELA | TACC3 | 4 | 1737405 | G | C | SNV | 0.132 | 20 | 152 |  | Category III |  |
| 3 | ELA | TP53 | 17 | 7577120 | C | T | SNV | 0.176 | 19 | 108 | p.R273H | Category II | T |
| 4 | Cancer | TACC3 | 4 | 1737405 | G | C | SNV | 0.117 | 72 | 618 |  | Category III |  |
| 4 | Cancer | TP53 | 17 | 7578474 | CG | C | Indel | 0.358 | 338 | 945 | p.P20fs*18 | Category I | ELA |
| 4 | ELA | TP53 | 17 | 7578474 | CG | C | Indel | 0.226 | 53 | 234 | p.P20fs*18 | Category I | T |
| 4 | ELA | CHEK2 | 22 | 29091840 | T | C | SNV | 0.13 | 12 | 92 | p.K373E | Category II |  |
| 5 | Cancer | ERBB4 | 2 | 212652758 | C | T | SNV | 0.172 | 33 | 192 |  | Category II | ELA |
| 5 | Cancer | APC | 5 | 112174833 | T | G | Indel | 0.172 | 51 | 296 | p.L1181* | Category I | ELA |
| 5 | Cancer | NRG1 | 8 | 32499413 | A | G | SNV | 0.172 | 39 | 227 |  | Category III | ELA |
| 5 | Cancer | NRG1 | 8 | 32617782 | G | A | SNV | 0.195 | 59 | 303 | p.V376I | Category II | ELA |
| 5 | Cancer | SMARCA4 | 19 | 11132542 | G | A | SNV | 0.208 | 154 | 741 | p.E920K | Category II | ELA |
| 5 | ELA | ERBB4 | 2 | 212652758 | C | T | SNV | 0.229 | 16 | 70 |  | Category II | T |
| 5 | ELA | APC | 5 | 112174833 | T | G | Indel | 0.189 | 18 | 95 | p.L1181* | Category I | T |
| 5 | ELA | NRG1 | 8 | 32499413 | A | G | SNV | 0.165 | 15 | 91 |  | Category III | T |
| 5 | ELA | NRG1 | 8 | 32617782 | G | A | SNV | 0.223 | 23 | 103 | p.V376I | Category II | T |
| 5 | ELA | SMARCA4 | 19 | 11132542 | G | A | SNV | 0.173 | 40 | 231 | p.E920K | Category II | T |
| 6 | Cancer | TP53 | 17 | 7577109 | AG | A | Indel | 0.198 | 85 | 430 | p.C277fs*66 | Category I | ELA |
| 6 | Cancer | TACC3 | 4 | 1737405 | G | C | SNV | 0.112 | 83 | 741 |  | Category III | ELA |
| 6 | ELA | TP53 | 17 | 7577109 | AG | A | Indel | 0.146 | 15 | 103 | p.C277fs*66 | Category I | T |
| 6 | ELA | TACC3 | 4 | 1737405 | G | C | SNV | 0.109 | 14 | 128 |  | Category III | T |
| 7 | ELA | PIK3CA | 3 | 178937755 | T | C | SNV | 0.106 | 25 | 235 |  | Category II |  |
| 7 | ELA | NRG1 | 8 | 32492781 | T | A | SNV | 0.111 | 29 | 261 |  | Category III |  |
| 7 | ELA | CHEK2 | 22 | 29090160 | G | C | SNV | 0.156 | 10 | 64 |  | Category III |  |
| 8 | Normal | NT5C2 | 10 | 104851274 | G | A | SNV | 0.426 | 20 | 47 |  | Category III | ELA |
| 8 | Cancer | CCDC6 | 10 | 61637836 | T | G | SNV | 0.101 | 25 | 247 |  | Category III | ELA |
| 8 | ELA | NOTCH2 | 1 | 120539500 | A | C | SNV | 0.121 | 44 | 364 |  | Category III |  |
| 8 | ELA | NOTCH2 | 1 | 120612013 | G | A | SNV | 0.126 | 66 | 523 | p.A3V | Category II |  |
| 8 | ELA | NOTCH2 | 1 | 120612014 | C | A | SNV | 0.129 | 66 | 510 | p.A3S | Category II |  |
| 8 | ELA | CCDC6 | 10 | 61637836 | T | G | SNV | 0.114 | 20 | 176 |  | Category III | T |
| 8 | ELA | NT5C2 | 10 | 104851274 | G | A | SNV | 0.533 | 24 | 45 |  | Category III | N |
| 8 | ELA | ERBB3 | 12 | 56481671 | T | C | SNV | 0.134 | 51 | 382 | p.S236P | Category II |  |
| 8 | ELA | IRAIN | 15 | 99192764 | T | C | SNV | 0.197 | 14 | 71 |  | Category II |  |
| 9 | Normal | NBPF20 | 1 | 145209350 | A | C | SNV | 0.1 | 96 | 958 |  | Category III | ELA |
| 9 | Normal | IRAIN | 15 | 99192764 | T | C | SNV | 0.103 | 14 | 136 |  | Category II | ELA |
| 9 | Cancer | APC | 5 | 112175639 | C | T | SNV | 0.468 | 103 | 220 | p.R1450fs*5 | Category I | ELA |
| 9 | ELA | NBPF20 | 1 | 145209350 | A | C | SNV | 0.108 | 56 | 518 |  | Category III | N |
| 9 | ELA | PIK3CA | 3 | 178937755 | T | C | SNV | 0.135 | 24 | 178 |  | Category II |  |
| 9 | ELA | APC | 5 | 112175639 | C | T | SNV | 0.161 | 40 | 249 | p.R1450fs*5 | Category I | T |
| 9 | ELA | NRG1 | 8 | 32476430 | G | T | SNV | 0.4 | 22 | 55 |  | Category III |  |
| 9 | ELA | IRAIN | 15 | 99192764 | T | C | SNV | 0.137 | 13 | 95 |  | Category II | N |
| 9 | ELA | CHEK2 | 22 | 29090160 | G | C | SNV | 0.157 | 14 | 89 |  | Category III |  |
| Chr.; Chromosome, Ref.; reference allele, Mut.; mutant allele, SNV; single nucleotide variant, Indel; insertion or deletion, MAF; mutant allele frequency. COSMIC; catalogue of somatic mutations in cancer. Coverage; sequence depth filtered monocular barcode after sequence, Category; Category I means frameshift indels or nonsense mutations, Category II means missense mutations, and Category III means synonymous changes or mutations located within introns. Origin means somatic mutations found in other tissues. | | | | | | | | | | | | | |
